# Climate Change and Climate Change Related Natural Disasters, and Maternal Mental Health: A Scoping Review of Socioeconomic Vulnerabilities

**DOI:** 10.1192/bjo.2025.10091

**Published:** 2025-06-20

**Authors:** Precious Jolugbo, Jennifer L. Barkin, Sanne van Rhijn

**Affiliations:** ^1^Imperial College London, London, United Kingdom; ^2^Mercer University School of Medicine, Georgia, USA; ^3^West London NHS Trust, London, United Kingdom

## Abstract

**Aims:** As the climate crisis escalates, pregnant women are increasingly exposed to extreme weather events, such as heatwaves and floods, which may lead to psychological distress and adverse mental health outcomes for both mothers and infants. This scoping review synthesizes research on the direct and indirect effects of climate change-related stressors on maternal mental health, identifying key trends, interventions, and mitigative strategies. Emphasis is placed on socioeconomic disparities in both high- and low-income countries, as these groups are disproportionately affected.

**Methods:** A systematic search was conducted to identify studies focusing on mental health, pregnancy (pre-, during, post-), and climate change, as defined in the AR6 Climate Change 2023 Synthesis Report, published up to October 2024. Data extraction included study design, population, interventions/exposures, outcomes, and socioeconomic implications. Only original articles and preprints in languages translatable to English were considered.

**Results:** The initial search retrieved 675 articles, of which 14 met the inclusion criteria. Two studies were from middle-income countries (Egypt and Turkey), while the remainder came from high-income countries (Australia, Canada, and the USA). The studies examined climate-related exposures, such as hurricanes, flooding, and extreme heat. Key findings indicate that acute exposure to high temperatures was associated with an increase in psychiatric emergency visits among pregnant women. Similarly, prenatal stress from natural disasters (e.g., hurricanes) was linked to higher levels of maternal mental health symptoms (e.g., depression, PTSD) and changes in infant temperament. Socioeconomic vulnerability played a critical role, with middle-income regions facing greater healthcare barriers, fewer mental health resources, and economic instability. Even in high-income regions, marginalized populations (e.g., Puerto Rico and the US Virgin Islands) experienced healthcare disruptions and prolonged recovery following climate disasters.

**Conclusion:** While the findings highlight the intersection of climate change and maternal mental health, several studies were limited by small sample sizes and reliance on self-reported data. A significant gap exists, as no studies specifically focused on maternal mental health in low-income countries affected by climate change were found during the literature search. Socioeconomic disparities strongly influenced mental health outcomes, underscoring the urgent need for equitable healthcare policies, financial support systems, and culturally adapted interventions. The review calls for the integration of climate resilience strategies into maternal healthcare and the strengthening of mental health infrastructure in low- and middle-income settings. Future research must prioritize longitudinal studies, policy-driven interventions, and targeted support for vulnerable populations.

